# Muzzling and Rabies

**Published:** 1899-06-24

**Authors:** 


### The Hospital

A Journal of

### The Medical Sciences and Hospital Administration.

Vol. XXVI.—No. 665.] Edited by Sir Henry Burdett, K.C.B., and Solomon C. Smith, M.D., M.R.C.P. [June 24, 1899.

### Muzzling and Rabies.

The Report for the year 1898, just published by the Board of Agriculture, is abundantly sufficient to show that, as regards the enforcement of muzzling as a preventive measure against rabies, Wisdom has been justified of her children. Only seventeen cases of the disease were detected in the whole of Great Britain during the year, as against 151 in 1897, 438 in 1896, and 672 in 1895. Fourteen of the seventeen cases occurred during the first half of the year, and two of the remainder prior to October. The single case subsequent to October occurred in a dog which had been completely isolated by order of the Board, on suspicion of having been in contact with a rabid dog. The figures manifestly support the hope that the disease, and its even more terrible concomitant, hydrophobia, will before long be completely extinguished in this country; but they do not permit the conclusion that this much-to-be-desired result has as yet been actually attained. The period of incubation cannot be said to have been positively determined, but it may unquestionably be a prolonged one; and, while it is a comparatively easy task to trace and destroy all bitten dogs in a rural neighbourhood, yet, when a case of rabies is detected in a thickly populated district, the most searching inquiries not infrequently fail to reveal the full amount of damage that has been done. In such districts, the extent to which the spread of infection can be checked by the destruction of the dogs which have been exposed to it is generally of a limited character, so that a considerable number of dogs may become affected, and some of these may pass on the disease to others, without the fact being discovered. In such localities, therefore, as the metropolis, or the manufacturing parts of the Midland Counties, of Lancashire, or of the West Riding of Yorkshire, it is difficult to determine how long the disease might maintain itself without any known cases being brought to the notice of the authorities; and the maintenance of muzzling orders for a long period in these places is essential if a thoroughly satisfactory result is to be secured. In the meantime, the work already accomplished has been such as entirely to justify the policy of the Board in confining the imposition of such orders to districts in which the disease was commonly prevalent, or in which it had recently appeared; to justify, in other words, that portion of this policy against which the largest amount of uninstructed criticism has been directed.

The experience of many months has now shown that the enforcement of a muzzling order, although it may be described as a matter of indifference to the dogs affected by it, is by no means a matter of indifference to the owners. An occasional puppy may be seen making endeavours to remove its muzzle, or a dog of mature years vainly striving to possess itself of some savoury morsel which appeals to the scavenging instincts of its race; but dogs as a rule seem no more afflicted by their muzzles than men by their hats or coats, or horses by their bits or harness. The owner, on the other hand, is called upon to take trouble in seeing that the dog does not leave home without the muzzle, that the muzzle is of an effective kind, that it is securely fastened, and so forth; with a liability to loss of time and the payment of penalties in case of neglect for which he may not really be responsible. It was therefore felt by the Board of Agriculture that the infliction of these inconveniences upon the owners of dogs must be restricted within the narrowest limits, and their very consideration in this respect has been made a weapon in the hands of silly and ignorant people. It has not been made so much a grievance that an owner in the West Riding should be compelled to muzzle his dog, as that an owner in the East Riding should be exempt from this necessity. The correctness of the view held by the Board has, however, been proved by experience, for, as the report quite correctly states, "it would have been impossible to have attained greater success if the Board had carried out the alternative suggestion" of an universal order. On the whole, the responsible authorities are able to say that "both the police and the public generally have heartily co-operated with the Board," that to this co-operation "the measure of success attained has been largely due," and that, "unless the disease be again introduced from without, the date of its final eradication is apparently not far distant." In the meanwhile, the importation of foreign dogs has been placed under the control of stringent regulations, such as are enforced in Norway and other countries in which rabies is unknown.

